# An integrated addictions nursing subspecialty to expand the opioid use disorder and substance use disorder workforce

**DOI:** 10.1192/j.eurpsy.2021.1521

**Published:** 2021-08-13

**Authors:** K. Williams, C. Selwyn, C. Elkins, S. Young, K. Pancione, M. Baker, Y. Getch

**Affiliations:** 1 College Of Nursing, University of South Alabama, Mobile, United States of America; 2 College Of Arts And Sciences, University of South Alabama, Mobile, United States of America; 3 College Of Education, University of South Alabama, Mobile, United States of America

**Keywords:** Integrated, Opioid, Substance use, addictions

## Abstract

**Introduction:**

In the U.S. approximately11.4 million misused prescription pain relievers; 2.1 million had an OUD in 2017. The Addictions Nursing Subspecialty was created to address this epidemic by expanding a workforce trained in OUD/SUD screening, treatment, and prevention. A curriculum was developed that included integrated/telehealth health care settings in medical and mental health provider shortage areas during their last nine months of training. Courses were developed and taught by aninterprofessional team of university faculty and informed by evidence-based guidelines/clinical competencies for effective OUD/SUD screening/prevention, assessment, treatment, and recovery. Courses were also offered as electives for nursing, clinical-counseling, social work, and other health science disciplines emphasizing an interdisciplinary approach to healthcare.

**Objectives:**

Expand the OUD/SUD trained workforce in areas with high OUD/SUD mortality rates and high mental health provider shortages emphasizing team-based integrated care and telehealth settings.

**Methods:**

Program curriculum was informed by evidence-based guidelines/clinical competencies for effective OUD/SUD screening/prevention, assessment, treatment, and recovery using integrated care. Competencies included: Core Competencies for Integrated Behavioral Health and Primary Care that have been set forth by the Center for Integrated Health Solutions, telehealth competencies outlined in the recommended competencies by the National Organization of Nurse Practitioner Faculties (NONPF), and Core Competencies for Addictions Medicine by the American Board of Addictions Medicine.

**Results:**

Approximately 11 students enrolled in courses received additions integrated/telehealth health care settings. Students responded positively to evaluations regarding timely feedback, unique approach (i.e. intrative content, short videos and discussions).

**Conclusions:**

The Addictions Nursing subspecialty will continue to be offered allowing enrollment for nurses twice a year.

